# Metabolic profiling and machine learning-based identification of key metabolites in 232 Huangjincha accessions

**DOI:** 10.1016/j.fochx.2026.104708

**Published:** 2026-10-28

**Authors:** Yang Zhao, Jiawei Yan, Furong Qu, Yibing Chen, Peidi Yang, Zongjie Wu, Yang Cheng, Wenmin Fan, Pengjie Wang, Zhen Liu, Saijun Li

**Affiliations:** a Tea Research Institute, Hunan Academy of Agricultural Science/Yuelushan Laboratory, Changsha 410125, Hunan, China; b College of Horticulture, Northwest A&F University, Yangling, Shaanxi 712100, China; c College of Information Engineering, Northwest A&F University, Yangling, Shaanxi 712100, China

**Keywords:** Huangjincha, Germplasm, Amino acid, Machine learning, Random Forest

## Abstract

Huangjincha (HJC), one of China's most important sexually propagated tea germplasm populations, exhibits extensive natural phenotypic and genotypic variation and has yielded six national elite cultivars and fourteen variety-protected cultivars. In this study, 35 metabolites were quantified by HPLC in 232 HJC accessions and 13 control cultivars. Statistical and multivariate analyses showed that catechins were the most abundant and variable metabolite class, whereas amino acids were significantly more abundant than in controls and exhibited the greatest stability. OPLS-DA identified 15 metabolites with VI*P* values above 1; except for 1,2,6-tris-*O*-galloyl-*β*-d-glucose and epicatechin, all were significantly more abundant in HJC. Ten machine-learning algorithms were used to construct metabolite-based discriminative models, with Random Forest performing best. Gini importance identified ten candidate discriminative metabolites, among which methionine, tyrosine, and proline were relatively stable and differed significantly from controls. These findings characterize HJC metabolism and support future validation of candidate markers for tea quality research.

## Highlights

• Amino acid metabolites exhibited low CV.
• GC, CG, leucine, theophylline, and theacrine showed high variability.
• Random Forest achieved the best performance in distinguishing HJC metabolites.
• Methionine, tyrosine, and proline as potential biomarkers of the population.

## 1 Introduction

Under long-term natural and artificial selection, populations of tea plant (*Camellia sinensis*) have developed stable traits while maintaining genetic diversity (Yu et al., 2020a; Lei et al., 2026). Cultivated populations, often derived from germplasm resources, are managed to preserve key agronomic and quality traits (Wang et al., 2020; Chen et al., 2023). The tea plant originated in China and possesses abundant germplasm resources, including wild relatives, local landraces, and cultivated populations (Yan et al., 2025; Yu et al., 2020b). The ‘Baojing Huangjincha’ [*Camellia sinensis* (L.) O. Kuntze ‘Baojing Huangjincha’] population, primarily seed-propagated in Baojing County, Hunan Province, is notable for early sprouting, high amino acid content, and fresh, brisk taste (Huang et al., 2022; Jiang et al., 2025; Yu et al., 2020b; Zhong et al., 2023). Six national-level cultivars, including ‘Baojing Huangjincha 1’, have been officially registered from this population, and fourteen additional cultivars have been granted plant variety protection. Since 2010, the HJC series of tea cultivars has been promoted in Hunan and surrounding provinces, with a total planting area exceeding 60,000 hm^2^ and an annual output value of billions of dollars (Liu et al., 2025).

Amino acids are important nitrogen-containing compounds in the tea plant and occur in both free and bound forms (Chen et al., 2020; Liu et al., 2025). They play important roles in growth, nitrogen metabolism, and the formation of tea quality traits, particularly freshness and sweetness (Huang et al., 2024; Li et al., 2025). More than twenty amino acids have been identified in tea leaves, among which theanine is a tea-specific amino acid, accounting for 40–60% of total free amino acids (Huang et al., 2022). Other free amino acids, such as methionine and aspartic acid, though less abundant, still contribute to flavor (Zhang et al., 2025). Bound amino acids, mainly in proteins or peptides, can be released during processing via hydrolysis, indirectly influencing tea quality. The total amino acid content of ‘Baojing Huangjincha 1’ peaks in spring (> 4%), higher than typical green teas (1–2%) (Li et al., 2025). Phenylalanine and tyrosine act as carbon‑nitrogen precursors for polyphenol and alkaloid biosynthesis. Phenylalanine, via phenylalanine ammonia-lyase (PAL), is converted to cinnamic acid and further to phenolic intermediates such as caffeic and gallic acids, which are key for flavonoid and catechin formation (Zhou et al., 2020). Gallic acid serves as an acyl donor to generate gallated catechins such as epigallocatechin gallate (EGCG) and epicatechin gallate (ECG), the primary chemical basis of tea's astringency (Chen et al., 2025; Shi et al., 2025).

Gallic acid and its esters, produced via the shikimate pathway, constitute a major part of tea phenolic acids and influence taste, enhancing mellowness and sweet lingering flavor (Li et al., 2017; Wang et al., 2025). Gallic acid serves as an acyl donor for galloylated catechins and can form esters with quinic acid or glucose (theogallin, *β*-glucogallin), acting as storage or activated forms for subsequent catechin galloylation (Wang et al., 2025). These compounds contribute to catechin structural elaboration, form complexes with alkaloids to modulate bitterness, and participate in polyphenol oxidation and condensation during processing, collectively shaping tea flavor and quality (Ye et al., 2022). Alkaloids, as nitrogen-metabolism products, are also major contributors to bitterness. Theobromine and theophylline are intermediates in purine alkaloid biosynthesis, with caffeine being the main end product and most abundant alkaloid in tea (Zhu et al., 2019). Caffeine can be oxidized to 1,3,7-trimethyluric acid or, in some germplasm, converted to theacrine, exhibiting distinct accumulation patterns (Zhang et al., 2020). These phenolic acids, catechins, and alkaloids not only contribute to key sensory and bioactive properties but also provide useful candidate biochemical indicators for evaluating metabolic variation in tea germplasm.

With advances in metabolomics, large-scale metabolic datasets are increasingly accessible but are high-dimensional and multivariate, often exhibiting complex nonlinear relationships that traditional methods cannot fully capture (Zhang et al., 2025). In contrast, machine learning approaches can efficiently identify informative features and model complex nonlinear patterns, offering improved performance in pattern recognition, feature selection, and classification prediction (Chen et al., 2023; Peng et al., 2023; Mansoor et al., 2025). Therefore, this study focused on 232 HJC accessions and 13 control tea cultivars. We selected 35 major non-volatile metabolites, including catechins, alkaloids, phenolic acids, and amino acids, as a targeted panel based on their known roles in tea taste and quality formation and the availability of authentic standards. Multiple binary classification algorithms were then applied to these metabolite profiles to characterize population-level metabolic variation. This analysis provides insights into the metabolic basis of HJC germplasm variation and, when integrated with machine learning, facilitates the identification of candidate discriminative metabolites that distinguish HJC from control tea cultivars within the current dataset.

## 2 Materials and methods

### 2.1 Sample collection

Tender shoots consisting of one bud and two leaves were collected from 232 Huangjincha (HJC) germplasm resources and 13 control tea cultivars between late March and mid-April 2024 at the experimental base of the Hunan Tea Research Institute, Gaoqiao Town, Changsha County, Hunan Province, China (113°19′E, 28°29′N). Healthy, morphologically uniform shoots without visible pest or disease damage were selected for sampling. The collected one bud and two leaves represent fresh leaf materials commonly used for green and black tea production. After harvesting, the samples were immediately placed in sterile centrifuge tubes and rapidly frozen in liquid nitrogen to minimize post-harvest metabolic changes. The samples were then freeze-dried using an FD5–6 vacuum freeze dryer (Jinximeng Instrument Co., Ltd., Beijing, China). The freeze-dried materials were ground into powder and used for subsequent metabolite analysis. The quantified metabolites included catechins, alkaloids, phenolic acids, and amino acids, which are major contributors to astringency, bitterness, freshness, and umami taste in tea. As freeze-dried leaf powder was used for extraction and quantification, all metabolite concentrations were expressed on a dry weight basis (mg/g DW).

The HJC germplasm resources used in this study were collected from their original distribution area during 2008–2010 and subsequently transplanted to a tea germplasm resource nursery. The control group comprised 13 representative non-HJC tea cultivars originating from different regions of China and representing diverse genetic backgrounds, processing suitability, plant types, leaf-size categories, and sprouting periods. All HJC accessions and control cultivars were maintained in the same germplasm resource nursery under the same cultivation, irrigation, and fertilization management conditions. This common cultivation environment helped reduce variation associated with differences in current cultivation and field management, although potential effects associated with original geographic origins and genetic backgrounds could not be completely excluded.

### 2.2 Determination of catechin and alkaloid components

Approximately 0.1 g of tea powder was weighed into a 50 mL centrifuge tube and extracted with 20 mL of 70% methanol (*v*/v). The mixture was vortexed and extracted in a water bath at 70 °C for 30 min. After cooling to room temperature, the extract was centrifuged at 8000 rpm for 10 min. The supernatant was filtered through a 0.45 μm membrane filter, and 1–1.5 mL of filtrate was transferred into an autosampler vial. Catechin and alkaloid components were analyzed using an LC-20 A HPLC system (Shimadzu, Kyoto, Japan) equipped with a Waters XSelect HSS T3 column (4.6 mm × 250 mm, 5 μm). The column temperature was maintained at 41 °C, the detection wavelength was set at 280 nm, the injection volume was 10 μL, and the flow rate was 1 mL min^−1^. The mobile phase consisted of solvent A (0.1% formic acid in water) and solvent B (acetonitrile). The gradient elution program was as follows: 0 min, 3% B; 13 min, 6% B; 17 min, 6% B; 26.5 min, 6.4% B; 30 min, 6.4% B; 35 min, 11% B; 40 min, 13% B; 50 min, 40% B; 53 min, 40% B; 56 min, 3% B; and 65 min, 3% B (Lu et al., 2009) (Fig. S3).

### 2.3 Determination of amino acid components

Approximately 0.1 g of tea powder was placed into a 15 mL centrifuge tube and extracted with 10 mL of boiling water. The mixture was incubated in a 100 °C water bath for 30 min. After cooling to room temperature, the extract was centrifuged at 6000 rpm for 10 min. The supernatant was filtered through a 0.22 μm aqueous membrane filter. For the preparation of standard solutions, 100 μL of amino acid standard solution was transferred into a 1.5 mL vial and mixed with 50 μL of internal standard solution. For sample preparation, 500 μL of the filtered extract was mixed with 50 μL of internal standard solution and transferred into an autosampler vial for analysis. Mobile phase A was prepared by dissolving 4.5 g Na₂HPO₄·12H₂O and 4.75 g Na₂B₄O₇·10H₂O in 1000 mL ultrapure water, adjusting the pH to 8.2 with 36% HCl, followed by filtration through a 0.45 μm membrane and ultrasonic degassing. Mobile phase B was prepared by mixing methanol, acetonitrile, and water (450: 450: 100, v: v: v) and degassing by ultrasonication. Amino acids were analyzed using an online derivatization method. Primary amino acids were derivatized with *O*-phthalaldehyde (OPA), whereas secondary amino acids were derivatized with fluorenylmethoxycarbonyl chloride (FMOC). The gradient elution program was as follows: 0 min, 5% B; 4.80 min, 9% B; 6.40 min, 9% B; 8.00 min, 15% B; 10.70 min, 26% B; 11.80 min, 26% B; 17.50 min, 40% B; 18.0 min, 53% B; 19.5 min, 53% B; 21.0 min, 100% B; 23.0 min, 100% B; 23.2 min, 5% B; and 25 min, 5% B. Chromatographic separation was performed on a Durashell-AA amino acid column with a column temperature of 50 °C and a flow rate of 2.0 mL min^−1^. Detection was carried out using a PDA detector at 338 nm for primary amino acids and 262 nm for secondary amino acids (Fig. S4). Theanine content was determined according to the Chinese national standard “Determination of theanine in tea-high-performance liquid chromatography method” (GB/T 23193–2017).

### 2.4 Reagents and standards

Reagents: HPLC-grade methanol and acetonitrile were purchased from Merck (Darmstadt, Germany). Disodium hydrogen phosphate dodecahydrate (Na₂HPO₄·12H₂O) was obtained from Kermel Chemical Reagent Co., Ltd. (Tianjin, China), and sodium tetraborate decahydrate (Na₂B₄O₇·10H₂O) was purchased from Macklin Biochemical Co., Ltd. (Shanghai, China). Standards. The reference standards of theobromine, caffeine, catechin (C), gallocatechin (GC), epicatechin (EC), epigallocatechin (EGC), epicatechin gallate (ECG), gallocatechin gallate (GCG), epigallocatechin gallate (EGCG), and catechin gallate (CG) were purchased from Sigma-Aldrich (St. Louis, MO, USA). Epigallocatechin-3-(3-*O*-methyl) gallate (EGCG3″Me) was obtained from Biotechnology Co., Ltd. (Sichuan, China). The standards of theophylline, theacrine, and 1,3,7-trimethyluric acid (1,3,7-TMU) were purchased from Yuanye Biotechnology Co., Ltd. (Shanghai, China). The standards of 1,2,6-tris-*O*-galloyl-*β*-d-glucose (1,2,6-TGG) and strictinin were obtained from Push Bio-Technology Co., Ltd. (Chengdu, China). The standard of theogallin was purchased from Tianzhi Biotechnology Co., Ltd. (Wuhan, China). Theanine standard was obtained from Desite Biotechnology Co., Ltd. (Chengdu, China). A mixed standard solution containing aspartic acid (Asp), glutamic acid (Glu), serine (Ser), histidine (His), glycine (Gly), threonine (Thr), arginine (Arg), alanine (Ala), tyrosine (Tyr), cystine (Cys-Cys), valine (Val), methionine (Met), isoleucine (Ile), phenylalanine (Phe), lysine (Lys), leucine (Leu), and proline (Pro) was purchased from Shimadzu (Kyoto, Japan).

### 2.5 Data preprocessing and construction of metabolite matrix

A metabolite matrix was constructed based on the quantitative data for 35 metabolites obtained from HPLC analysis, with the concentration of each metabolite used as an input feature for model construction. Binary classification labels were assigned according to sample type, with HJC samples labeled as “1” and control (CK) samples labeled as “0”. The 35 metabolites quantified by HPLC were defined independently of the HJC/CK group labels and were included as input features in the machine-learning analyses. No supervised feature pre-selection based on group-wise differences or model-derived feature importance was performed before model training. Machine learning analyses were conducted using Python version 3.13.5.

### 2.6 Construction and training of the HJC classification models

To discriminate HJC samples from the control group, ten machine learning models were constructed and implemented, covering commonly used algorithms including generalized linear models, distance- or kernel-based models, probabilistic graphical models, tree-based models, and neural networks. Specifically, the generalized linear models included Logistic Regression (both the standard version and a stochastic gradient descent-based implementation); distance- or kernel-based models included K-Nearest Neighbors (KNN) and Support Vector Machine (SVM); probabilistic graphical models included Gaussian Naive Bayes and Quadratic Discriminant Analysis (QDA); tree-based models included Random Forest (RF), Extra Trees, and Gradient Boosting Decision Tree (GBDT); and the neural network model was a Multilayer Perceptron (MLP). All models were implemented using the scikit-learn package in Python. Before model training, z-score standardization was applied to Logistic Regression, SVM, and MLP, whereas the other models were trained using unstandardized feature values. Model parameters were set either to predefined values or scikit-learn default settings (Chen et al., 2023; Peng et al., 2025; Wang et al., 2025; Lei et al., 2025).

To ensure reliable model evaluation, stratified sampling was used to randomly split the dataset into a training set (80%) and a test set (20%), maintaining the original class proportions between HJC and CK samples. The full dataset contained 232 HJC samples and 13 CK samples. After stratified splitting, the training set contained 186 HJC and 10 CK samples, while the test set contained 46 HJC and 3 CK samples. All models were trained on the training set, and the random seed was fixed at 42 to ensure reproducibility (Mansoor et al., 2025). Five-fold stratified cross-validation was further performed on the training set, with each validation fold containing 37–38 HJC samples and 2 CK samples.

### 2.7 Model testing and evaluation

The test set was used to evaluate model performance. Confusion matrices were constructed based on model predictions, and the numbers of true positives (TP), true negatives (TN), false positives (FP), and false negatives (FN) were calculated. Using these basic statistics, multiple classification metrics were computed, including accuracy, precision, recall (or sensitivity), specificity, F1-score, and the area under the receiver operating characteristic curve (AUC). The formulas for these metrics are as follows:

(1) $$\mathrm{Accuracy}=\frac{\mathrm{TP}+\mathrm{TN}}{\mathrm{TP}+\mathrm{TN}+\mathrm{FP}+\mathrm{FN}}$$

(2) $$\mathrm{Precision}=\frac{\mathrm{TP}}{\mathrm{TP}+\mathrm{FP}}$$

(3) $$\mathrm{Sensitivity}=\mathrm{Recall}=\frac{\mathrm{TP}}{\mathrm{TP}+\mathrm{FN}}$$

(4) $$\mathrm{Specificity}=\frac{\mathrm{TN}}{\mathrm{TN}+\mathrm{FP}}$$

(5) $$\mathrm{Balanced Accuracy}=\frac{\mathrm{Sensitivity}+\mathrm{Specificity}}{2}$$

(6) $$F1-\mathrm{score}=\frac{2\times \left(\mathrm{Precision}\times \mathrm{Recall}\right)}{\text{Precision}+\text{Recall}}$$

Accuracy represents the overall proportion of correctly predicted samples; precision reflects the proportion of correctly predicted positive samples among all predicted positives; recall (sensitivity) indicates the model's ability to identify positive samples; specificity measures the model's ability to identify negative samples; and F1-score, the harmonic mean of precision and recall, provides a comprehensive assessment of model performance, especially for imbalanced data. All metrics range from 0 to 1, with values closer to 1 indicating better classification performance.

To assess model robustness, five-fold stratified cross-validation was performed on the training set. In each iteration, four folds were used for model training and the remaining fold for validation, with each fold serving as the validation set once. The mean and standard deviation (mean ± SD) of the performance metrics across the five folds were calculated. The test set was excluded from model training and cross-validation and reserved for final evaluation.

To further assess the potential effects of class imbalance, Logistic Regression, support vector machine (SVM), Random Forest, and Extra Trees were evaluated with and without balanced class weights using repeated stratified three-fold cross-validation (30 repeats) on the training set. The same data splits were used for both weighting settings. Model performance was assessed using AUC, accuracy, balanced accuracy, sensitivity, specificity, precision, and F1-score. Mean and standard deviation across validation folds were used for descriptive comparison.

Based on the cross-validation results, the best-performing models were selected for subsequent feature importance analysis. Feature importance was assessed using mean decrease in Gini and permutation importance. Mean decrease in Gini quantifies the contribution of each feature to impurity reduction during tree splitting, whereas permutation importance evaluates the decrease in model performance after the values of a feature are randomly permuted.

To evaluate the stability of candidate metabolite selection, feature importance was assessed across 90 model fits generated by repeated stratified three-fold cross-validation within the training set. For each fit, the 35 metabolites were ranked according to Gini importance and permutation importance, and their frequencies of occurrence among the top 10 features were calculated. The consistency between the two ranking methods was further evaluated using top-10 overlap, the Jaccard index, and Spearman rank correlation. This analysis assessed feature-selection stability within the current dataset rather than external generalizability.

To assess the overall statistical significance of the classification models, a permutation test was performed using the training set. Class labels were randomly permuted 1000 times, and model performance was evaluated using five-fold stratified cross-validation. The pooled out-of-fold AUC was used as the primary performance metric. To account for model selection, the maximum AUC across the 10 models was recorded for each permutation to construct a null distribution. The empirical P value was calculated as follows:

(7) $$P=\frac{\left(b+1\right)}{\left(B+1\right)}$$

where b is the number of permutations in which the maximum AUC was equal to or greater than the observed maximum AUC, and B is the total number of permutations (1000). The test set was excluded from this analysis.

### 2.8 Multivariate and statistical analyses

The coefficient of variation (CV) for each metabolite was calculated as follows:

(8) $$\mathrm{CV}\left(\%\right)=\frac{s}{\bar{x}}\times 100$$

where s and $\bar{x}$ represent the standard deviation and mean metabolite abundance, respectively.

The diversity index (*H*′) was calculated as follows:

(9) $$H^{\prime }=-\sum P_{i}\ln\left(P_{i}\right)$$

where *P*_*i*_ represents the relative abundance of the *i*-th component. The standardized metabolite intensity index, denoted as SI_norm_, was then calculated as follows:

(10) $${\mathrm{SI}}_{\mathrm{norm}}=\frac{\mathrm{CV}\left(\%\right)/100}{H^{\prime }+\left|\mathrm{Skewness}\right|+1}$$

This index integrates metabolite variability, diversity, and distributional asymmetry. Skewness and kurtosis were computed using the built-in functions in Excel 2021. One-way analysis of variance (ANOVA) was performed using IBM SPSS Statistics 25, followed by multiple comparisons with Tukey's HSD test to evaluate significant differences in metabolite abundances among samples (*p* < 0.05). To further explore overall metabolic differences and classification features among samples, K-means clustering, principal component analysis (PCA), and orthogonal partial least squares discriminant analysis (OPLS-DA) were conducted using the Metware Cloud online platform (https://cloud.metware.cn/) for the HJC germplasm and control samples. Variable importance in projection (VIP) scores were calculated for all metabolites. Data visualization, including pie charts, bar plots, and violin scatter plots, was performed using Origin 2021. Receiver operating characteristic (ROC) curves were used to calculate the Youden index, which was then employed to determine the optimal classification threshold (cut-off value), according to the formula:

(11) Youden index=Sensitivity+Specificity−1

## 3 Results and discussion

### 3.1 Analysis of the HJC germplasm population and metabolites

The HJC germplasm population is a genetically diverse, sexually propagated tea population with considerable breeding potential. Among its desirable traits, high amino acid content represents one of the most valuable quality characteristics for breeding and research (Zhong et al., 2023; Jiang et al., 2025; Liu et al., 2025). To date, six nationally recognized tea cultivars have been successfully developed from the HJC population (Fig. 1A). Using HPLC, a total of 35 metabolites belonging to four major classes were quantified in the HJC germplasm and control samples (Table S1). Analysis of metabolite composition revealed that catechins were the predominant compounds in the HJC population, accounting for 52.83% of total quantified metabolites, with EGCG being the most abundant, followed by ECG, indicating that catechins constitute the main chemical component of this tea population. Alkaloids accounted for 17.11% of total quantified metabolites, primarily contributed by caffeine and theobromine. Phenolic acids and amino acids accounted for 15.18% and 14.88% of the total quantified metabolites, respectively, with the highest contents observed for theogallin acid and theanine (Fig. 1B). Compared with the control samples, the HJC population exhibited significantly lower total catechin and phenolic acid contents, whereas amino acid contents were significantly higher, and alkaloid levels showed no significant difference (Fig. 1C). These results indicate that, despite a catechin-dominated chemical background, the HJC population displays a quality advantage characterized by amino acid enrichment, providing an important chemical basis for its fresh and brisk taste.

**Fig. 1:**
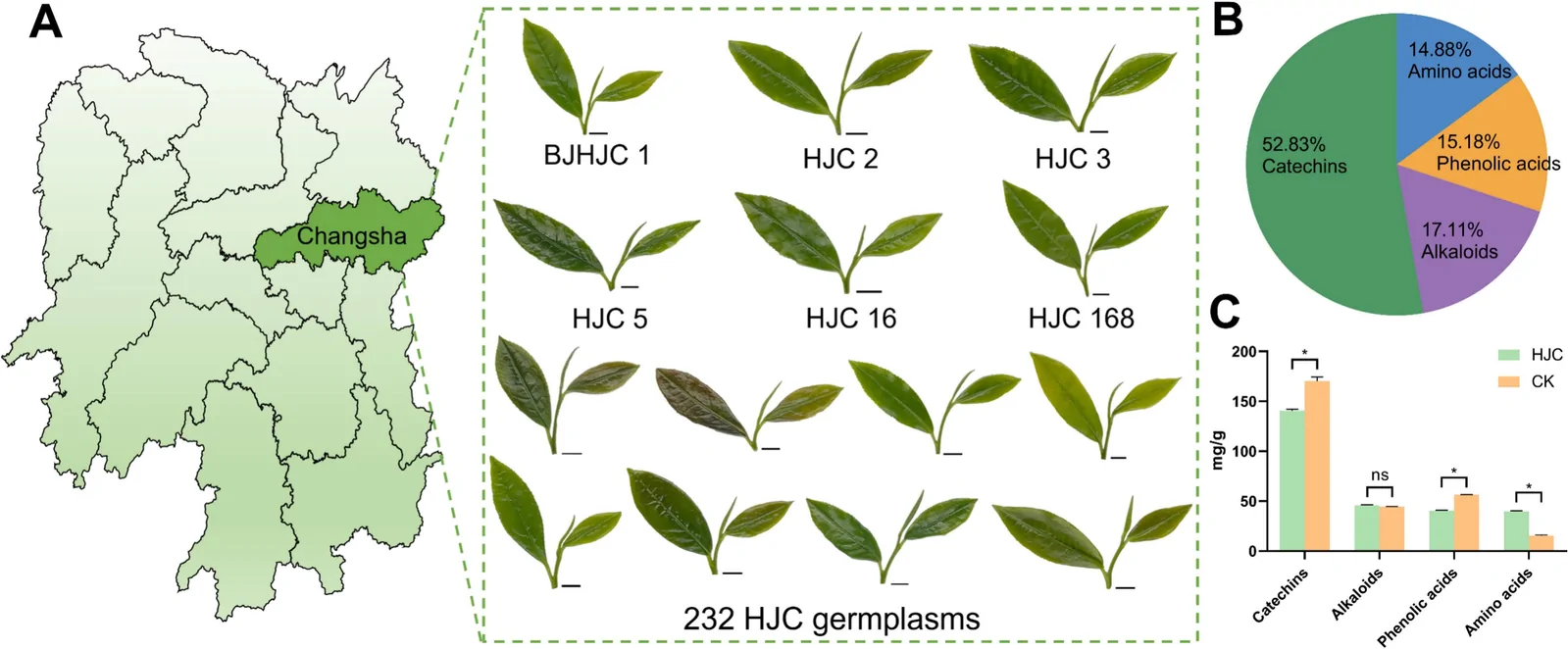
Representative leaf phenotypes and major metabolites of the HJC germplasm population. (A) Leaf phenotypes of HJC germplasm resources. Six accessions represent released HJC cultivars, while the remaining accessions are HJC germplasm resources. Scale bar = 1 cm. (B) Relative proportions of catechins, alkaloids, phenolic acids, and amino acids among the 35 quantified metabolites in 232 HJC germplasm samples. (C) Comparison of metabolite contents between HJC samples and controls (HJC: *n* = 232; CK: *n* = 13). Asterisks indicate significant differences (*p* < 0.05); ns, not significant.

Statistical analysis of the 35 individual metabolites in the HJC germplasm population revealed pronounced variation in metabolite abundances at the population level (Table 1). Most metabolites exhibited coefficients of variation (CV) ranging from 20% to 80%, whereas gallocatechin (GC), catechin gallate (CG), leucine, theophylline, and theacrine showed CV values exceeding 100%, indicating substantial phenotypic diversity in secondary metabolite accumulation within the HJC population. Among amino acids, theanine was the most abundant and displayed relatively low variability, suggesting its stable enrichment across the population. In contrast, other free amino acids exhibited considerable individual variation and skewed distributions, reflecting potentially more complex genetic regulation. Moreover, the skewness and kurtosis of most metabolites significantly deviated from normality, and normality tests confirmed that their abundance distributions did not conform to the normal assumption, further highlighting the high metabolic heterogeneity of the sexually propagated HJC population (Table S2). These results indicate that individual metabolite profiling supports the potential for trait mining in HJC germplasm. When examined by metabolite class, catechins exhibited substantial variability (CV = 17–167%, SI-norm = 0.03–0.12), suggesting that catechins are a primary source of metabolic differentiation and quality variation in the HJC population. Among them, EGCG was relatively stable, whereas CG and GC were less consistent. In contrast, amino acids showed the highest stability (CV = 14–47%, SI-norm = 0.02–0.05), reflecting their role as metabolically stable components within the population. Therefore, amino acids may serve as potential core indicators of metabolic homeostasis in HJC.

**Table 1:** Summary statistics of metabolite contents in the HJC germplasm population and comparison with control cultivars.

Metabolite	Mean ± SD (mg/g)		CV% (HJC)	*H*′ (HJC)	Skewness (HJC)	SI-norm (HJC)
	HJC (*n* = 232)	CK (*n* = 13)				
C	1.19 ± 0.47a	1.34 ± 0.61a	39.04	5.39	4.84	0.03
EC	4.53 ± 1.40b	7.11 ± 2.68a	30.78	5.40	0.57	0.04
GC	0.59 ± 0.97b	2.05 ± 1.14a	162.44	4.92	7.29	0.12
EGC	13.18 ± 5.23a	1.35 ± 0.61b	39.67	5.37	1.28	0.05
CG	0.60 ± 0.99a	0.61 ± 0.57a	167.07	4.91	8.11	0.12
ECG	25.44 ± 5.18b	29.43 ± 8.73a	20.36	5.43	0.34	0.03
GCG	1.11 ± 0.42b	1.55 ± 1.53a	37.44	5.38	0.77	0.05
EGCG	91.40 ± 16.16b	107.70 ± 17.63a	17.68	5.43	−0.16	0.03
EGCG3”Me	2.47 ± 1.26a	2.81 ± 2.59a	50.83	5.32	0.76	0.07
Theobromine	5.91 ± 1.87a	2.81 ± 1.60b	31.56	5.40	0.22	0.05
Theophylline	0.03 ± 0.05a	0.03 ± 0.03a	165.08	4.73	3.47	0.18
Theacrine	0.17 ± 0.25a	0.16 ± 0.18a	149.83	4.99	6.31	0.12
Caffeine	34.33 ± 3.66a	32.92 ± 5.25a	10.67	5.44	1.82	0.01
1,3,7-TMU	1.30 ± 0.73a	0.07 + 0.04b	55.92	5.30	1.04	0.08
Strictinin	3.77 ± 3.14b	8.35 ± 3.43a	83.32	5.14	1.63	0.11
Theogallin	39.71 ± 11.72b	49.23 ± 12.66a	29.52	5.39	−0.67	0.04
1,2,6-TGG	0.67 ± 0.52b	7.18 ± 6.16a	77.27	5.20	2.09	0.09
Theanine	23.37 ± 4.37a	9.28 ± 4.04b	18.71	5.43	−0.08	0.03
Asp	3.48 ± 0.51a	1.35 ± 0.33b	14.69	5.44	−0.44	0.02
Glu	4.17 ± 0.61a	1.88 ± 0.39b	14.61	5.44	−0.50	0.02
Ser	0.89 ± 0.16a	0.58 ± 0.28b	18.52	5.43	−0.27	0.03
His	1.63 ± 0.95a	0.24 ± 0.46b	58.52	5.28	1.14	0.08
Gly	0.05 ± 0.01a	0.03 ± 0.00b	25.40	5.42	2.08	0.03
Thr	0.33 ± 0.07a	0.23 ± 0.05b	21.14	5.42	−0.51	0.03
Arg	3.37 ± 1.26a	0.91 ± 0.38b	37.39	5.38	0.51	0.05
Ala	0.91 ± 0.30a	0.26 ± 0.09b	32.73	5.39	0.17	0.05
Tyr	0.14 ± 0.05a	0.02 ± 0.01b	37.28	5.38	0.68	0.05
Cys-Cys	0.50 ± 0.12a	0.36 ± 0.10b	24.79	5.42	0.20	0.04
Val	0.09 ± 0.02a	0.03 ± 0.02b	24.38	5.42	0.52	0.04
Met	0.22 ± 0.11a	0.03 ± 0.00b	47.32	5.35	2.20	0.06
IIe	0.02 ± 0.01b	0.03 ± 0.01a	62.58	5.30	3.12	0.07
Phe	0.06 ± 0.01a	0.04 ± 0.01b	22.58	5.42	−0.29	0.03
Lys	0.09 ± 0.03a	0.03 ± 0.01b	28.14	5.41	0.09	0.04
Leu	0.07 ± 0.09a	0.02 ± 0.01b	138.81	5.21	14.62	0.07
Pro	0.19 ± 0.05a	0.04 ± 0.02b	27.05	5.41	2.63	0.03

Note: Mean ± SD represents the mean and standard deviation of metabolite contents in 232 HJC samples and 13 CK samples. Different lowercase letters within the same row indicate significant differences between HJC and CK based on Tukey's HSD test at *p* < 0.05. CV% represents the coefficient of variation of each metabolite in the HJC population. *H*′ represents the Shannon diversity index of metabolite abundance distribution in the HJC population. SI-norm represents the normalized metabolite intensity index in the HJC population. Skewness represents the degree of asymmetry in the distribution of metabolite abundance within the HJC population.

Violin plots combined with scatter and box plots were used to illustrate the concentration distributions and individual variability of metabolites within the HJC population. EGCG exhibited a broad distribution, with the highest mean abundance and the largest variation range (37.87–135.33 mg/g) among catechins, significantly exceeding other catechin components (Fig. 2A). Elevated levels of EGCG may be associated with defense responses and antioxidant capacity in tea plants and may serve as a key chemical basis for tea bitterness and astringency (Wang et al., 2025). ECG and EGC showed intermediate mean levels with noticeable individual variation, yet their overall abundance was substantially lower than that of EGCG. Other catechins, including catechin (C), epicatechin (EC), gallocatechin gallate (GCG), and epigallocatechin-3-(3-*O*-methyl) (EGCG3”Me), were present at relatively low concentrations with minor inter-individual differences. GC and CG showed low mean abundances, suggesting limited overall accumulation in HJC, potentially increasing only in specific individuals (Fig. 2A). Among alkaloids, caffeine was markedly higher than other components, representing the predominant purine alkaloid in the HJC population. Theobromine, strictinin, and 1,3,7-trimethyluric acid were present at lower mean levels than caffeine but still accumulated to a detectable extent, while other alkaloid components remained at low levels across the population (Fig. 2B). Among free amino acids, theanine displayed the highest mean concentration and is an important contributor to the fresh and brisk taste of HJC. Its distribution was relatively broad (11.17–33.39 mg/g), indicating significant inter-individual variation. Glutamic acid, aspartic acid, and arginine were present at moderate levels with detectable accumulation, whereas proline, tyrosine, and methionine were generally low and exhibited limited population-level variability (Fig. 2C). Theogallin, an important gallic acid derivative in tea, is closely involved in the biosynthesis of galloylated catechins such as EGCG and ECG (Zhu et al., 2025). Its mean abundance was second only to EGCG and displayed substantial variation across the population (0.17–68.56 mg/g), indicating marked population-level differentiation (Fig. 2D). Notably, theogallin itself has low bitterness and a fresh, mild taste, which may partially mitigate the bitterness of tea. In contrast, 1,2,6-TGG was present at low concentrations (Fig. 2D).

**Fig. 2:**
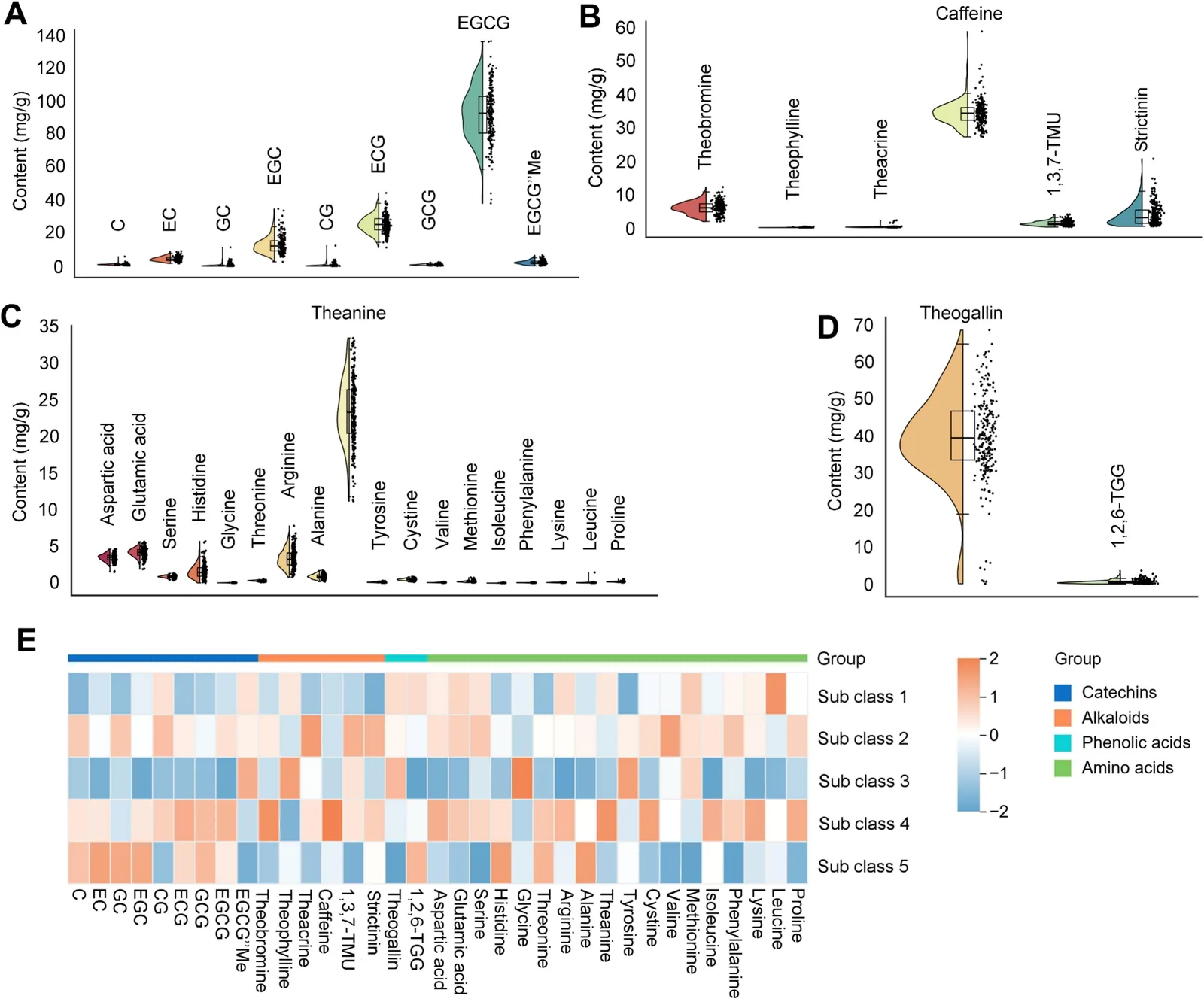
Relative abundances of 35 metabolites in the HJC population. (A) Concentrations of nine catechin components (mg/g). (B) Concentrations of six alkaloid components (mg/g). (C) Concentrations of 18 amino acid components (mg/g). (D) Concentrations of two phenolic acid components (mg/g). (E) Heatmap of mean standardized accumulation levels of individual metabolites across the five HJC subclasses.

Based on standardized metabolite data, K-means clustering classified the 232 HJC germplasm samples into five subclasses (Fig. S1). Subclass 1 comprised 64 samples and was characterized by relatively high phenolic acid scores, followed by catechins. Subclass 2 included 74 samples and showed high scores for both catechins and phenolic acids, representing a catechin- and phenolic-acid-enriched group. Subclass 3 consisted of 49 samples and displayed a more pronounced phenolic acid-dominant pattern. Subclass 4 comprised 37 samples and exhibited higher catechin scores, with phenolic acids at intermediate levels, representing a catechin-enriched group. Subclass 5 included only 8 samples and was characterized by markedly elevated catechin levels, forming a high-catechin subclass. To further identify the specific metabolites distinguishing different subclasses, we visualized a heatmap of the mean standardized accumulation levels of individual metabolites in each subclass (Fig. 2E). The results showed that different catechin components exhibited differentiated relative accumulation patterns among subclasses. Among them, non-galloylated catechins, such as C, EC, GC, and EGC, showed relatively high accumulation levels in Subclass 5, whereas galloylated catechins, such as CG, ECG, GCG, and EGCG, were relatively enriched in Subclass 4. Among phenolic acid compounds, theogallin showed a relatively high accumulation level in Subclass 3 and may be one of the main contributors to the phenolic acid-enriched pattern of this subclass. In addition, multiple metabolites in Subclass 4 showed relatively high accumulation levels, including theobromine, caffeine, and theanine. Most amino acid compounds showed relatively high accumulation patterns in Subclass 1, Subclass 2, and Subclass 4, such as aspartic acid, glutamic acid, serine, and lysine. Overall, the HJC subclasses differed not only in the overall trends of the four major metabolite categories but also in the relative accumulation patterns of specific individual metabolites.

### 3.2 Differential metabolite analysis based on the HJC population

To better characterize the metabolic features of the HJC population, 13 commonly cultivated tea varieties were selected as controls for subsequent analyses. PCA was performed using quantitative data of 35 metabolites. As shown in Fig. 3A, the first two principal components explained a total variance of 38.47%. Along PC1, HJC and control samples were well separated, with HJC exhibiting significantly higher scores than controls, although a few HJC samples were positioned outside the main cluster, which did not affect the overall intergroup differences. Along PC2, control samples displayed significantly higher scores than the HJC population. Building on the PCA results, OPLS-DA was conducted to further distinguish the metabolic profiles. The model showed effective separation between the two groups, with R^2^X = 0.493, R^2^Y = 0.743, and Q^2^ = 0.667. Both R^2^Y and Q^2^ were statistically significant (*p* < 0.05), supporting that the OPLS-DA model captured meaningful metabolic differences within the current dataset (Fig. 3B and C). However, given the limited number of control cultivars, the generalizability of the OPLS-DA results requires further validation. Based on this model, 15 metabolites with VIP scores greater than 1 were identified (Fig. 3D). Among the differential metabolites, only 1,2,6-tris-*O*-galloyl-*β*-d-glucose (7.14 mg/g) and EC (7.06 mg/g) showed higher mean abundances in the control group, whereas the remaining metabolites were more abundant in the HJC population.

**Fig. 3:**
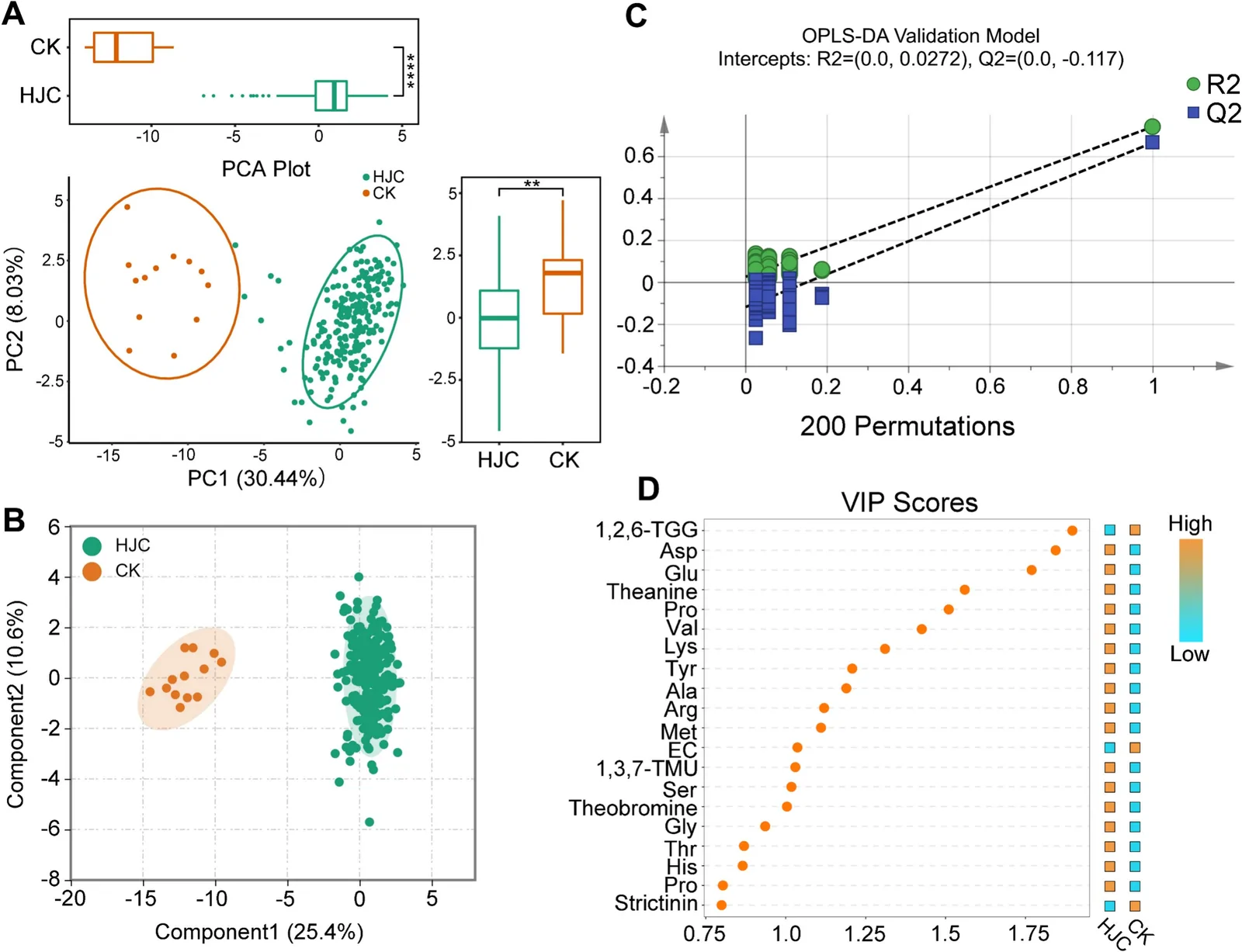
Metabolite differences and key metabolite identification in the HJC population. (A) PCA scatter plots of HJC and control (CK) samples. (B) OPLS-DA scatter plots of HJC and CK samples. (C) Permutation test of the OPLS-DA model with 200 iterations. (D) VIP scores of differential metabolites (left) and a heat map of their relative abundances (right), (HJC: *n* = 232；CK: *n* = 13).

### 3.3 Development of multiple machine learning models

To evaluate whether metabolite profiles could distinguish HJC from control samples, a machine-learning workflow was constructed using the 35 quantified metabolites as input features and sample type as the classification label. The workflow included data preprocessing, stratified train/test splitting, multi-model training, and cross-validation-based model evaluation (Fig. 4A–C). Ten machine-learning models were compared, including linear, kernel-based, probabilistic, distance-based, neural-network, and tree-based models. Their predictive performance and stability were evaluated using multiple metrics, including AUC, accuracy, balanced accuracy, precision, recall, F1-score, sensitivity, and specificity (Table S3).

**Fig. 4:**
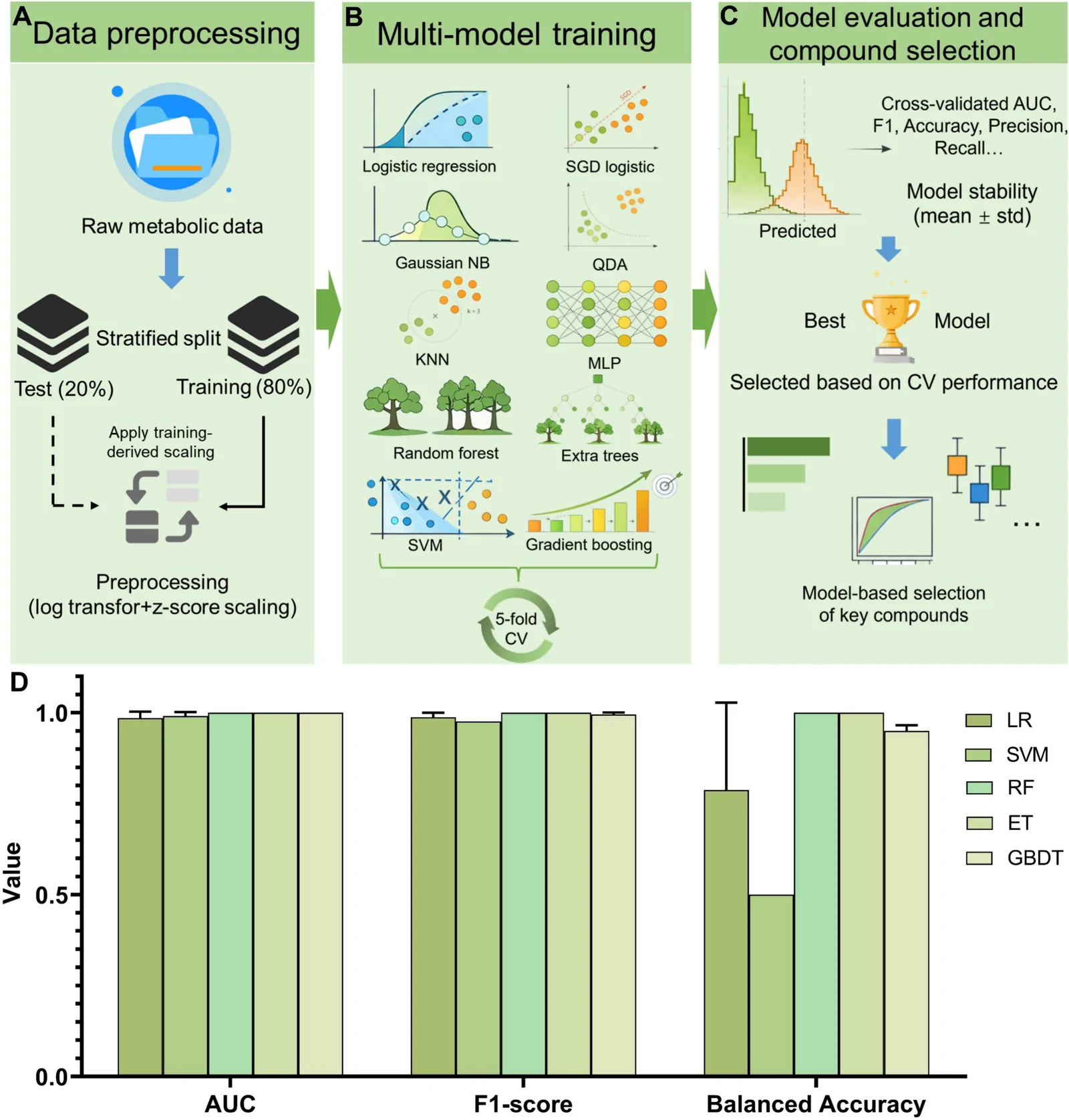
Workflow for multi-type machine learning modeling, evaluation, and key metabolite selection based on metabolomics data. (A) Stratified sampling of the original metabolite data into training (80%) and test (20%) sets. (B) Training of 10 machine learning models on the training set and evaluation via 5-fold stratified cross-validation. (C) Selection of optimal models based on cross-validated performance metrics, including AUC, F1-score, accuracy, precision, and recall. (D) Performance of selected machine learning models evaluated by 5-fold cross-validation, values represent mean ± standard deviation obtained from 5-fold cross-validation.

The results revealed substantial variation among models across performance metrics (Table S3). Random Forest and Extra Trees achieved perfect cross-validation scores for AUC, accuracy, balanced accuracy, and F1-score, while Gaussian Naive Bayes also showed strong performance under the internal evaluation framework. However, these results reflect performance within the current dataset. Gradient Boosting also performed well, with a mean AUC of 1.000, F1-score of 0.997, and balanced accuracy of 0.950. In contrast, Logistic Regression and Multilayer Perceptron showed high AUC and F1-scores but lower balanced accuracy, indicating less consistent recognition of the two classes. Although Support Vector Machine (SVM) achieved a mean AUC of 0.995, its balanced accuracy was only 0.500, with a specificity of 0.000, reflecting poor recognition of control samples. SGD-logistic, K-Nearest Neighbors, and Quadratic Discriminant Analysis exhibited comparatively lower performance in some evaluation metrics.

Based on classification performance, negative class recognition, and model stability, five representative models were selected for further statistical analysis (Fig. 4D). Logistic Regression and SVM were chosen as representatives of linear and kernel-based models, respectively, serving as non-tree model comparisons. Random Forest, Extra Trees, and Gradient Boosting represented the best-performing tree-based models. Random Forest and Extra Trees achieved perfect scores (AUC, F1-score, and Balanced accuracy = 1.000), and Gradient Boosting maintained relatively stable classification performance. These tree-based models were selected for subsequent feature importance analysis because they enabled a consistent comparison of Gini-based and permutation-based importance across different tree ensemble algorithms. Logistic Regression and SVM, despite high AUC values, were inferior to tree-based models in terms of balanced accuracy and stability. Accordingly, Random Forest, Extra Trees, and Gradient Boosting were selected for subsequent feature selection and identification of key metabolites. To further evaluate the overall significance of model performance, a permutation test was conducted using the training set with 1000 random label permutations. The observed maximum cross-validation AUC across the 10 models was 1.000, whereas the mean and 95th percentile of the permutation-based maximum AUC distribution were 0.633 and 0.759, respectively. The global max-statistic permutation test yielded *P* = 0.000999, indicating that the observed classification performance was significantly higher than expected under the permuted-label null distribution (Table S4).

To assess the potential effects of class imbalance, four classification algorithms were evaluated with and without balanced class weights using repeated stratified three-fold cross-validation on the training set (Table S5). The effects of class weighting varied across algorithms. Logistic Regression showed an increase in balanced accuracy from 0.994 to 1.000 and in control specificity from 0.987 to 1.000. In contrast, Extra Trees and Random Forest showed minor decreases in these metrics after class weighting. For SVM, balanced accuracy increased from 0.830 to 0.841 and control specificity increased from 0.659 to 0.681, although control identification remained less accurate than that of the other models. Overall, class weighting did not consistently improve classification performance across algorithms, highlighting the importance of considering class-sensitive metrics when evaluating models trained on imbalanced datasets.

### 3.4 Analysis based on tree ensemble models

First, the contributions and stability of different metabolite classes were systematically evaluated across three tree-based models at the chemical category level. Assessment using mean Gini importance, permutation importance, and normalized permutation importance indicated that amino acids consistently scored highest across all three metrics, with normalized permutation importance exceeding 2.5, suggesting that amino acids contribute most stably to the models (Fig. S2). Amino acids not only serve as fundamental building blocks for tea growth and development but are also closely associated with tea flavor and the biosynthesis of volatile compounds (Yu et al., 2024). These results underscore the significance of the high amino acid content characteristic of the HJC population. In contrast, catechins and phenolic acids scored lower across all metrics, indicating a limited impact on model stability. Overall, amino acids were the major contributors to model feature importance at the chemical category level and may contribute to the metabolic differences between HJC and control samples. However, their relative contributions should be interpreted in the context of the current dataset and the importance metrics used.

Feature importance was further evaluated based on three high-performing tree models: Random Forest, Extra Trees, and Gradient Boosting. In the Random Forest model, methionine had the highest Gini importance, indicating its key role in distinguishing samples, followed by tyrosine and proline, while theanine exhibited the lowest score and contributed minimally to classification (Fig. 5A). Methionine also displayed the highest permutation importance, whereas all other metabolites had near-zero permutation scores (Fig. 5D). In the Extra Trees model, aspartic acid, glutamic acid, and lysine showed high importance scores, with methionine and tyrosine also relatively significant (Fig. 5B). Only aspartic acid, lysine, methionine, tyrosine, and 1,3,7-trimethyluric acid had notable permutation importance scores, with aspartic acid being the highest; other metabolites contributed minimally (Fig. 5E). In the Gradient Boosting model, only methionine, 1,3,7-trimethyluric acid, and tyrosine exhibited appreciable Gini and permutation importance scores (Fig. 5C and F). Considering the results from the three tree-based models and both importance metrics, methionine emerged as a prominent candidate discriminative metabolite, while tyrosine, proline, aspartic acid, and 1,3,7-trimethyluric acid also showed potential discriminatory value. However, the importance rankings varied across models and evaluation methods, suggesting that the relative contributions of individual metabolites were not fully consistent. These metabolites should therefore be regarded as candidate discriminative metabolites for distinguishing HJC from CK samples.

**Fig. 5:**
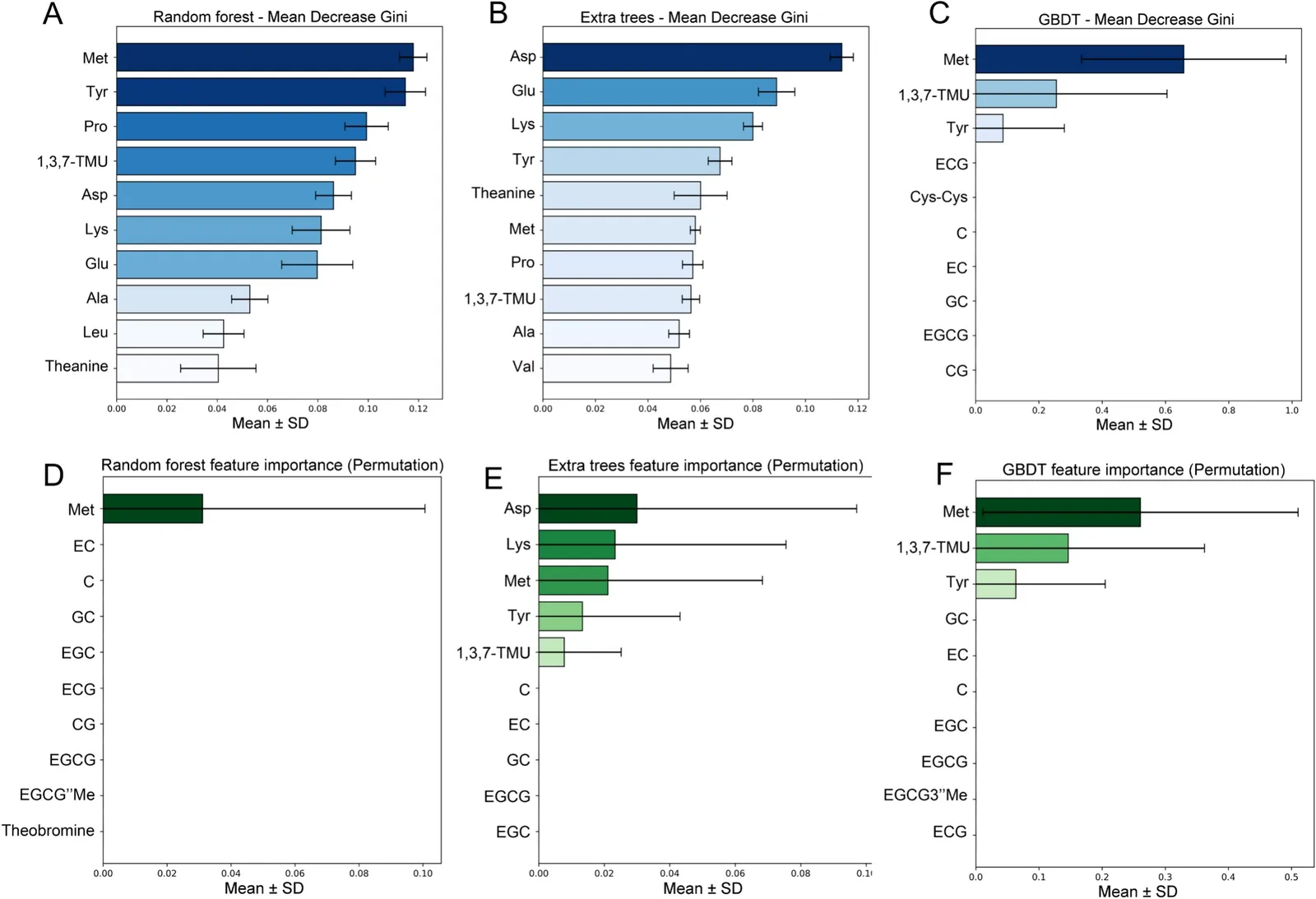
Comparison of metabolite feature importance derived from Random Forest, Extra Trees and Gradient Boosting models. (A-C) Top 10 metabolites ranked by mean decrease in Gini importance in Random Forest, Extra Trees and Gradient Boosting models, respectively. (D—F) Top 10 metabolites ranked by permutation importance in Random Forest, Extra Trees and Gradient Boosting models, respectively.

Feature importance stability was further evaluated across 90 model fits using repeated stratified three-fold cross-validation within the training set. Based on Gini importance, methionine, 1,3,7-Trimethyluric acid, and tyrosine were consistently ranked among the top 10 features in all 90 fits, while aspartic acid, proline, and lysine were selected in 98.9%, 98.9%, and 97.8% of the fits, respectively. However, agreement between Gini and permutation importance was limited. The two methods shared an average of 1.40 features in their respective top 10 rankings, with a mean Jaccard index of 0.132. The mean Spearman rank correlation was 0.349 among the 44 fits in which it could be calculated. These findings indicate that several metabolites showed stable Gini-based rankings, whereas their importance was less consistently supported by permutation analysis.

### 3.5 Identification of key metabolites in the HJC population using the random Forest model

Although the three tree-based models achieved AUC values of 1.0 in the original model evaluation, the stability of individual metabolite features required further assessment. The top 10 candidate discriminative metabolites were initially selected based on mean Gini importance from the Random Forest model. Mann–Whitney *U* tests were then used to compare metabolite abundances between the HJC and control groups, and ROC analysis was performed to assess the discriminatory ability of individual metabolites. The Youden index was used to determine the optimal cut-off value for each metabolite. The selected candidates included methionine, tyrosine, proline, 1,3,7-trimethyluric acid, aspartic acid, lysine, glutamic acid, alanine, leucine, and theanine. All 10 metabolites showed individual AUC values greater than 0.98 in the original analysis, with methionine reaching an AUC of 1.0. However, because candidate selection and discriminatory assessment were conducted using the same dataset, these AUC values should be interpreted as exploratory estimates rather than evidence of independently validated marker performance. Boxplots showed significantly higher abundances of these metabolites in HJC than in the control group (*p* < 0.05). However, additional stability analysis revealed limited agreement between Gini and permutation importance, indicating that the importance rankings of individual metabolites depended partly on the evaluation method. Therefore, these metabolites are considered candidate discriminative markers identified in the current dataset, and their robustness requires further validation.

Methionine and tyrosine play important roles in the biosynthesis of tea volatile aroma compounds, particularly tyrosine, which interacts with phenylalanine to promote the accumulation of floral and fruity volatiles (Xu et al., 2020). As shown in Fig. 6A and B, the levels of methionine and tyrosine in HJC were significantly higher than those in the control. Proline content is relatively stable in the HJC germplasm (Fig. 6C), and it can improve tea flavor and mouthfeel by regulating nitrogen availability, thereby influencing amino acid content and protein synthesis, and consequently affecting the freshness and briskness of the tea infusion (Huang et al., 2018). Proline also showed significant group differences (Fig. 6C). These metabolites exhibited strong discriminatory ability in the original dataset; however, their feature importance rankings were not equally supported by Gini and permutation importance. Their potential roles in HJC-associated metabolic characteristics therefore warrant further investigation. 1,3,7-trimethyluric acid, aspartic acid, lysine, and glutamic acid exhibited moderate discriminative ability (Fig. 6D, E, F and G). 1,3,7-trimethyluric acid, a purine metabolite, is a key intermediate in caffeine biosynthesis and directly affects bitterness and astringency in tea (Zhu et al., 2025). Aspartic acid, lysine, and glutamic acid are involved in nitrogen metabolism and protein synthesis in tea plants, contribute to hormone biosynthesis and stress resistance, and serve as key amino acids associated with tea freshness and delicate aroma (Ruan et al., 2019). Theanine is the most abundant amino acid in the HJC germplasm and a tea-specific amino acid, serving as an important nitrogen source (Fig. 6J). Theanine not only enhances the freshness of the tea infusion but also reduces its bitterness (Guo et al., 2018; Lei et al., 2026). Alanine and leucine showed similar physiological roles but had relatively lower discriminatory power between HJC and control samples (Fig. 6H and I). These results suggest that amino acid metabolism may contribute to the metabolic characteristics of the HJC population. These metabolites may serve as candidate biochemical indicators for distinguishing HJC samples within the current dataset. The identified metabolites may provide a candidate material basis for the development of metabolic markers in subsequent breeding research and may also inform future studies on HJC-related metabolic characteristics and quality traits.

**Fig. 6:**
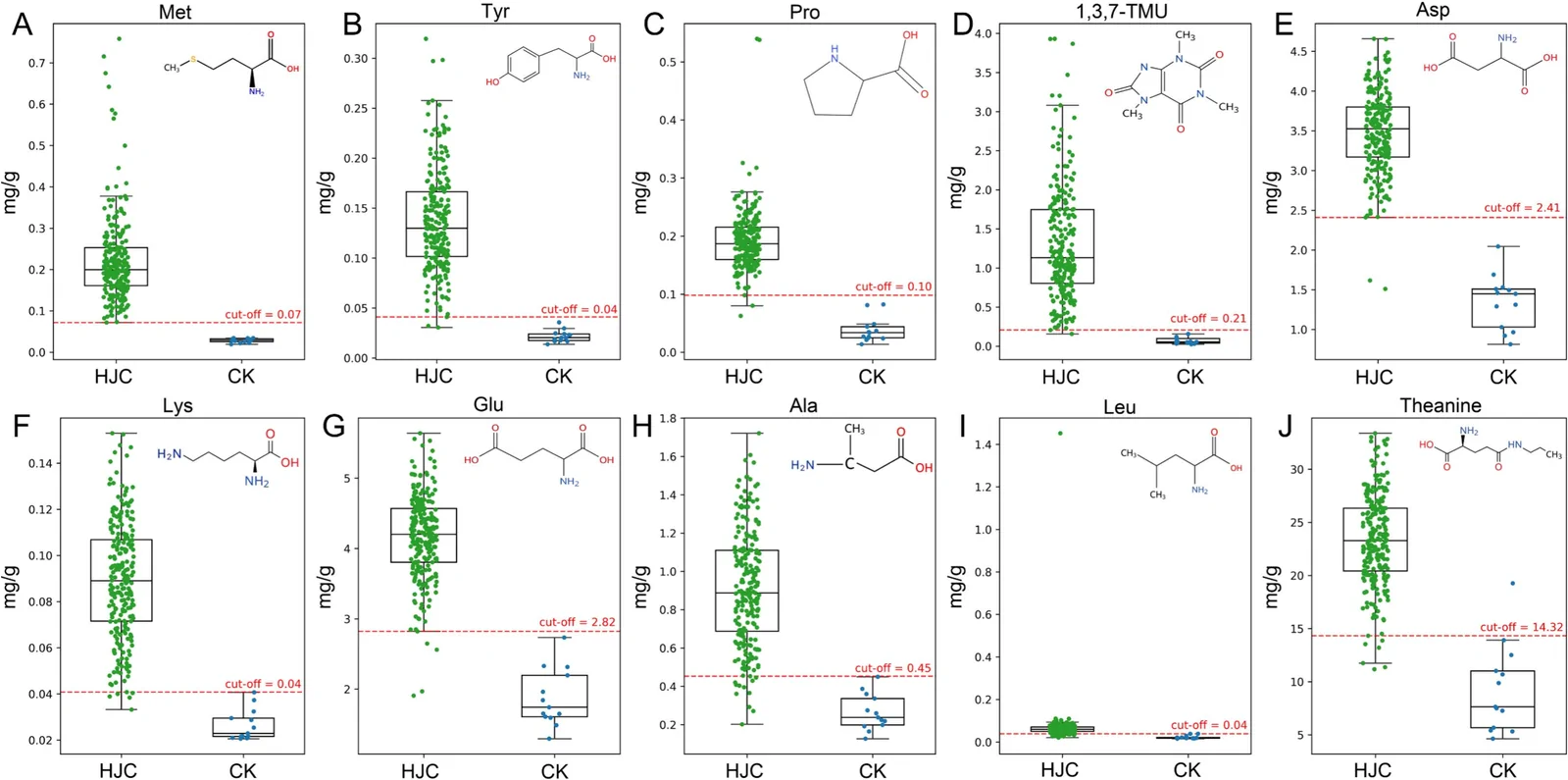
Concentration differences of the top 10 metabolites selected by the Random Forest model between HJC and control samples. (A) Methionine (Met); (B) Tyrosine (Tyr); (C) Proline (Pro); (D) 1,3,7-Trimethyluric acid (1,3,7-TMU); (E) Aspartic acid (Asp); (F) Lysine (Lys); (G) Glutamic acid (Glu); (H) Alanine (Ala); (I) Leucine (Leu); (J) Theanine. Red dashed lines indicate the cut-off values. (For interpretation of the references to color in this figure legend, the reader is referred to the web version of this article.)

This study identified metabolic features that distinguished HJC accessions from the control cultivars within the current dataset and established a preliminary framework for characterizing HJC-like metabolic profiles using metabolite profiling and machine learning. The identified metabolites provide candidate biochemical markers for subsequent evaluation of HJC-related metabolic characteristics. However, their applicability to unknown tea samples and their potential use in germplasm screening or authenticity assessment remain to be established through independent validation. Compared with single-compound discrimination, this multi-metabolite approach provides a more comprehensive biochemical fingerprint and may offer a basis for subsequent quality assessment and germplasm screening.

However, the control group comprised only 13 cultivars, compared with 232 HJC accessions. Although all materials were maintained under the same cultivation and field management conditions, differences in geographic origins, genetic backgrounds, and potentially plant type, leaf morphology, and sprouting phenology may have contributed to the observed metabolic differences. Therefore, these differences cannot be attributed exclusively to HJC identity, and the classification performance and candidate metabolites should be interpreted within the context of the current dataset. Future studies should include larger and more representative control populations, independent validation populations, samples collected across different years, and commercial tea products to assess the robustness and broader applicability of the candidate metabolites. Furthermore, integrating these metabolites with sensory evaluation and other tea quality-related traits will help clarify their relationships with specific quality attributes and assess their potential as indicators of tea quality. Such research may provide a candidate material basis for developing metabolic markers in subsequent tea breeding research.

## 4 Conclusion

In this study, we analyzed 232 Huangjincha germplasm accessions by integrating quantitative data on amino acids, catechins, alkaloids, and phenolic acids and applied machine learning approaches to identify candidate metabolites that distinguish HJC accessions from control cultivars. Amino acids were generally more abundant in the HJC group, whereas catechins showed considerable variation within the population. Among the evaluated classification models, Random Forest showed high apparent discriminative performance within the current dataset. Based on feature importance analyses, ten candidate discriminative metabolites were identified: methionine, tyrosine, proline, 1,3,7-trimethyluric acid, aspartic acid, lysine, glutamic acid, alanine, leucine, and theanine. Methionine, tyrosine, and proline were prominent candidates for distinguishing HJC from control samples, although the importance rankings varied across models and evaluation methods. These findings provide a quantitative characterization of the HJC metabolome and a candidate material basis for further investigation of metabolic markers in tea breeding research. Future studies should validate these candidate metabolites using independent and representative tea germplasm populations and integrate metabolite profiles with sensory evaluation and other tea quality-related traits to assess their potential as indicators of tea quality and clarify their relevance to specific quality attributes.

## CRediT authorship contribution statement

**Yang Zhao:** Writing – original draft, Software, Methodology, Investigation, Formal analysis, Data curation, Conceptualization. **Jiawei Yan:** Writing – original draft, Methodology, Conceptualization. **Furong Qu:** Methodology, Investigation. **Yibing Chen:** Methodology, Investigation. **Peidi Yang:** Methodology, Investigation. **Zongjie Wu:** Methodology. **Yang Cheng:** Methodology. **Wenmin Fan:** Funding acquisition. **Pengjie Wang:** Project administration, Funding acquisition. **Zhen Liu:** Writing – review & editing, Project administration, Funding acquisition. **Saijun Li:** Writing – review & editing, Project administration, Funding acquisition, Conceptualization.

## Funding

This work was supported by the Hunan Provincial Agricultural Science and Technology Innovation Fund (2025CX43), the 10.13039/501100001809National Natural Science Foundation of China (32472794), the National Tea Industry Technology System (CARS-19), and the Hunan Provincial Agricultural Science and Technology Innovation Fund (2025CX91).

## Declaration of competing interest

The authors declare that they have no known competing financial interests or personal relationships that could have appeared to influence the work reported in this paper.

## Data Availability

The scripts used for machine learning analysis, including normalization, model training, cross-validation, performance evaluation, and feature importance analysis, together with the input metabolite matrix and fixed random seed information, have been deposited in a public GitHub repository: https://github.com/yjwT258/HJC-Metabolite_ML.

## Appendix A Supplementary data

Figure S1. K-means clustering of samples based on concentrations of catechins, alkaloids, phenolic acids, and amino acids (M1-M4 represent catechins, alkaloids, phenolic acids, and amino acids, respectively). Fig. S2. Feature importance and stability of metabolite classes across three tree-based models. Fig. S3. Separation chromatograms of standard and sample at 280 nm. Fig. S4. Separation chromatograms of standard and sample at 338 and 262 nm. Table S1. Summary of metabolite data for all samples in this study. Table S2. Complete statistical characteristics and differential comparison of 35 metabolites between HJC germplasm and control cultivars. Table S3. Performance comparison of all machine learning models evaluated by 5-fold cross-validation. Table S4. Permutation test results for the ten classification models. Table S5. Class-weighting sensitivity analysis of four classification models.

Supplementary material 1 Supplementary figures and chromatograms.

Supplementary material 2 Metabolite data and statistical analyses.
