# Supplementary material for: Metabolic profiling and machine learning-based identification of key metabolites in 232 Huangjincha accessions
Source: Food Chem X. 2026 Oct 28;40:104708. doi: 10.1016/j.fochx.2026.104708 (PMC13742232; doi:10.1016/j.fochx.2026.104708)
Supplement: Supplementary file 1 [file mmc1.docx]

Supplementary figure:

Figure S1. K-means clustering of samples based on concentrations of catechins, alkaloids, phenolic acids, and amino acids (M1-M4 represent catechins, alkaloids, phenolic acids, and amino acids, respectively)

Figure S2. Feature importance and stability of metabolite classes across three tree-based models. (A) Average Gini importance of catechins, alkaloids, phenolic acids, and amino acids across Random Forest, Extra Trees, and Gradient Boosting Decision Tree models.

(B) Normalized permutation stability, representing the frequency with which each metabolite class appears among the top 10 features after random permutation.

(C) Permutation stability, showing the raw frequency of each metabolite class ranked among the top 10 features.

Figure S3. Separation chromatograms of standard and sample at 280 nm. 1, theogallin. 2, gallocatechin. 3, theobromine, 4, theophylline. 5, 1,3,7-trimethyluric acid. 6, epigallocatechin. 7, catechin. 8, theacrine. 9, caffeine. 10, strictinin. 11, epicatechin. 12, epigallocatechin gallate. 13, gallocatechin gallate. 14, 1,2,6-tris-*O*-galloyl-*β*-D-glucose. 15, epigallocatechin-3-(3-*O*-methyl). 16, epicatechin gallate. 17, catechin gallate.

Figure S4. Separation chromatograms of standard and sample at 338 and 262 nm. 1, aspartic acid. 2, glutamic acid. 3, serine. 4, histidine. 5, glycine. 6, threonine. 7, arginine. 8, alanine. 9, theanine. 10, tyrosine. 11, cystine. 12, valine. 13, methionine. 14 isoleucine. 15, phenylalanine. 16, lysine. 17, leucine. 18, proline.
